# The Acute Effects of Moderate-Intensity Aerobic Exercise on Core Executive Functions in Healthy Older Adults: A Systematic Review

**DOI:** 10.3390/life15020230

**Published:** 2025-02-05

**Authors:** Erdem Çakaloğlu, Hidayet Suha Yüksel, Fatma Neşe Şahin, Özkan Güler, Erkal Arslanoğlu, Bade Yamak, Mert Aydoğmuş, Onur Mutlu Yaşar, Alper Cenk Gürkan, Mehmet Söyler, Levent Ceylan, Hamza Küçük

**Affiliations:** 1 Faculty of Sport Sciences, Ankara University, Golbasi, Ankara 06830, Türkiye; ecakaloglu@ankara.edu.tr (E.Ç.); hsyuksel@ankara.edu.tr (H.S.Y.); nesesahin@ankara.edu.tr (F.N.Ş.); oguler@ankara.edu.tr (Ö.G.); 2 Faculty of Sport Sciences, Sinop University, Sinop 57000, Türkiye; erkaloglu@sinop.edu.tr; 3 Yasar Dogu Faculty of Sport Sciences, Ondokuz Mayis University, Samsun 55200, Türkiye; bade.tekbas@omu.edu.tr; 4 Department of Coaching Education, Hasan Dogan Faculty of Sport Sciences, Karabuk University, Karabuk 78050, Türkiye; aydogmus1978mert@gmail.com; 5 Faculty of Health Sciences, Izmir Demokrasi University, Izmir 35140, Türkiye; onurmutluyasar@gmail.com; 6 Department of Vocational School of Health Services, Gazi University, Ankara 06830, Türkiye; 7 Social Sciences Vocational High School, Cankiri Karatekin University, Cankiri 18200, Türkiye; mehmetsoyler@karatekin.edu.tr; 8 Faculty of Sport Sciences, Hitit University, Corum 19030, Türkiye; leventceylan17@hotmail.com

**Keywords:** cognitive functions, cognitive flexibility, elderly, inhibition, working memory

## Abstract

There is growing interest in examining acute effects of exercise on cognitive functions and neurocognitive outcomes. These behavioral and neurocognitive outcomes have been most frequently investigated in healthy young individuals, but relatively few studies have examined healthy older adults. This study aimed to systematically review the effects of acute moderate-intensity aerobic exercise (MIAE) on core executive functions, including inhibition, working memory, and cognitive flexibility, in healthy older adults. A database search of PubMed, Scopus, and Web of Science was conducted using a systematic search strategy. Acute MIAE interventions assessing core executive functions using randomized or stratified controlled trials investigating healthy older adults were reviewed. Eleven studies were identified, and the behavioral results from all included studies revealed that acute MIAE can improve core executive functions in healthy adults. However, incompatible results were observed in activated areas of the prefrontal cortex following MIAE in older adults. The limited number of studies investigating the effects of MIAE on core executive functions in older healthy adults with moderate overall quality restricts the conclusions. Therefore, more robust quality studies using neuroimaging techniques to investigate core executive functions, especially working memory and cognitive flexibility, are needed to explain the neural and behavioral mechanisms.

## 1. Introduction

The term “executive functioning” (EF) encompasses a set of high-level cognitive processes that enable individuals to engage in goal-directed actions [1,2]. EF is one of the main components of human cognition and skill acquisition. EF is involved in maintaining attentional control, especially when a thought or behavior contradicts habits, impulses, or desires. It has a close relationship with neural activity in the prefrontal cortex (PFC) [3,4,5], including the dorsolateral prefrontal cortex (DLPFC) [6], ventrolateral prefrontal cortex (VLPFC) [7], frontopolar area (FPA) [8], and inferior frontal gyrus (IFG) [9,10] as well as inferior parietal lobule [11,12]. EF does not represent a unitary/single construct; however, it can be conceptualized as comprising the core EFs (e.g., inhibition/interference, working memory/updating, cognitive flexibility/switching-shifting) and higher-level EFs (e.g., decision making, problem-solving/planning) [1]. Inhibition, which is the central domain of EF, represents the ability to obstruct automatic or impulsive responses and act more purposely by controlling attention and behavior [13]. Working memory refers to holding and updating/manipulating the current mental content with new information. Lastly, cognitive flexibility involves quickly and flexibly switching between changing demands or priorities of tasks or mental sets [1].

A growing body of evidence indicates that EF is sensitive to age-related alterations in brain structure and function [14,15,16,17,18]. However, many studies have confirmed the broad-spectrum benefits of aerobic exercise in overcoming age-related declines and maintaining a healthy life for older adults [19,20,21]. The interest in the chronic effects of moderate-intensity aerobic exercise (MIAE) on older populations has increased over the last 2 decades [22,23,24]. Firth et al. [25] suggested in their meta-analysis that aerobic exercise has positive effects on delaying and/or protecting hippocampal volume reduction in healthy older adults. Also, Hendrikse et al. [26] suggested that engaging in higher exercise levels is associated with better hippocampal structure and function in adults. Moreover, chronic MIAE has been reported to increase the volumes of gray and white matter in the PFC and temporal cortex [27]. Therefore, MIAE has been suggested as a potentially effective intervention for enhancing cognition among younger individuals [28,29,30] and older adult [31,32] populations.

Over the past decade, there has been a notable increase in research interest aimed at investigating the acute effects of MIAE on EF and neurocognitive outcomes. The effects of acute MIAE on EF cannot be explained by a single neural mechanism. Some of the neural mechanisms underlying these effects may be summarized as follows: efficient attentional resource allocation to task-dependent stimuli, improved conflict detection, and increased activation in specific areas of the PFC (e.g., DLPFC, VLPFC, FPA) following exercise [6,30,33,34,35,36,37,38,39,40]. However, these neurocognitive outcomes and mechanisms have been most frequently examined in healthy young populations; accordingly, a clear consensus on this issue has not yet been reached in healthy older adults. Chang et al. [30] focused on the dose–response relationship between exercise duration and EF in young adults and suggested that 20 min of MIAE had the most beneficial effects on EF test performance compared with 10 and 45 min. Yanagisawa et al. [6] concluded that after acute MIAE, EF-related test performances were enhanced, and higher activations in the left DLPFC were observed in young adults. Mehren et al. [33] observed that acute MIAE was more beneficial than high-intensity aerobic exercise for EF and that MIAE resulted in more activated EF-related brain areas in young adults. In addition, acute MIAE has positive effects on EF, specifically by facilitating the allocation of attentional and neural resources and enhancing conflict detection in young adults [37].

The relationships between exercise and cognition in older adults have typically been investigated in the context of chronic exercise, with less attention being paid to the effects of acute exercise [41]. Three systematic review studies examined the acute effects of exercise on cognitive functions in older adults [42,43,44]. Although Chang et al. [42] reported a positive and small overall effect (g = 0.181; 95% confidence interval [CI]; 0.073, 0.290) and a more beneficial effect for cognitive testing following exercise (g = 0.108; 95% CI; 0.069–0.147) than after a delay (g = 0.103; 95% CI; 0.035–0.170) in which cognitive assessments were administered after at least 15 min of exercise cessation, these results are independent of exercise intensity, exercise type, cognitive domain, and population (impaired samples included). On the other hand, Ludyga et al. [43] reviewed the acute effects of MIAE on EF in participants aged over 50 years and reported positive and moderate effects (g = 0.67; *p* < 0.001) when reaction time was considered as the dependent variable. More recently, McSween et al. [44] investigated the immediate effects of acute aerobic exercise on cognitive function and reported that nearly all of the studies observed improved performance in at least one cognitive test parameter.

However, the aforementioned authors [42,43,44] examined only behavioral findings of various intensities of exercise and different cognitive function domains. In particular, Ludyga et al. [43] included studies with participants aged 50 years or older, which may affect the results that volume decline per year in the brain structures has been reported [45,46]. Therefore, it would be difficult to draw clear conclusions from these reviews. In addition, when interest in the link between behavioral and neural outcomes following exercise has increased over the last decade, this topic has not been studied in previous reviews. Considering the growing body of literature on the acute effects of MIAE on EF in older adults, there is a need for an updated review of recent studies, including neurophysical measurements. Therefore, the purpose of this review was to systematically review the effects of acute MIAE on specific domains of EF, including inhibition, cognitive flexibility, and working memory in healthy older adults.

## 2. Materials and Methods

### 2.1. Search Strategy and Study Selection

This systematic review was carried out following the PRISMA (Preferred Reporting Items for Systematic Reviews and Meta-Analyses) statement detailing the preferred items to report in a systematic review [47]. The protocol was not registered. Three electronic databases (PubMed, Scopus, and Web of Science) were screened until 29 September 2024, and studies published after 2010 were considered. The search focused on covering the areas of acute MIAE, core EF, and older adults using the following key terms and strings, either singly or in combination: (acute OR single-session OR single-bout) OR (moderate exercise OR moderate-intensity aerobic exercise OR aerobic exercise) AND (executive function OR inhibition OR cognitive flexibility OR working memory) AND (healthy AND (older adults OR elderly)) (Supplementary File S1).

**Figure 1:**
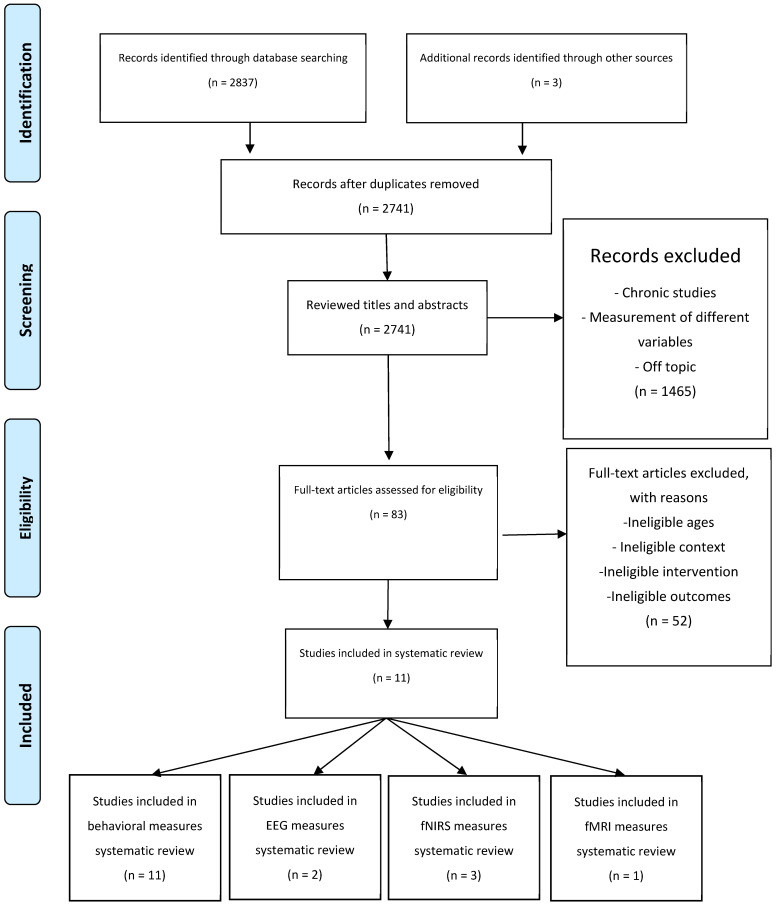
Preferred reporting items for Systematic Reviews and Meta-Analyses flow of studies through the review.

The searching, identification, screening, and quality assessment were performed by 2 study team members (E.Ç. and H.S.Y.) independently. Initially, the same team members screened the titles to determine whether the articles were related to the research purpose. Second, the studies were screened through their abstracts if they met the inclusion and exclusion criteria. Third, a secondary exclusion process was performed using full-text articles if the inclusion criteria were unclear in the abstracts. Finally, the reference lists of the full-text articles were checked to identify other potentially eligible papers. If there was any disagreement, 2 researchers discussed it, and if an agreement could not be achieved, inclusion was decided by a third study team member (F.N.Ş.). A summary of the data collection process is presented in Figure 1.

### 2.2. Figures, Tables, and Schemes

#### 2.2.1. The Type of Study

Acute or single-session design-evaluating interventions in randomized or stratified controlled trials were reviewed.

#### 2.2.2. Type of Participants

Studies examining healthy older adults aged >55 years were reviewed.

#### 2.2.3. Type of Interventions

Studies that performed acute aerobic exercises between 40% heart rate reserve (HRR) to 60% HRR, 50% to 70% VO2 max, 64% to 77% HR max, or the Borg rating of perceived exertion (RPE) scores 13 to 15 were included.

#### 2.2.4. Outcome Measures

The primary outcome measure was at least one core EF (inhibition, cognitive flexibility, and working memory) assessed within 45 min of exercise cessation; this was reviewed. In addition, neurophysiological outcome measures, including EEG, fNIRS, and fMRI, assessing core EF were included.

#### 2.2.5. Exclusion Criteria

Studies with one of the following criteria were excluded: (1) non-randomized, uncontrolled, cross-sectional studies, single-case studies, qualitative studies, reviews, and non-intervention studies; (2) studies with participants under 55 years old or with mental or neurological disorders or chronic medical illnesses; (3) studies investigating low or high-intensity aerobic exercise or physical activity, other types of exercise interventions (e.g., balance, strength, stretching), exercises also have cognitive demands (e.g., exergame), anaerobic exercise, concurrent exercise, combined exercise, or cognitive training, assessing the cognitive outcomes only before exercise performed, investigating the chronic effects of exercise with multiple sessions of exercise, exercise combined with supplements or a pharmacological treatment; (4) studies assessing the other cognitive functions except for core EF or administration of the cognitive test after 45 min of exercise cessation.

#### 2.2.6. Data Extraction

The Cochrane Consumers and Communication Review Group’s data extraction standardized protocol was used to extract (1) study characteristics, including author(s), title, and year of publication; (2) study design; (3) participant information, such as sample size, age, and sex; (4) description of the exercise intervention, including types of exercise, intensity, and duration; (5) cognitive outcomes, including task, domain of EF, and timing of test administration; and (6) neurophysiological outcomes, including EEG, fNIRS, and fMRI measurements.

After the initial stages of inclusion, the quality of the full-text studies was evaluated using the Physiotherapy Evidence Database (PEDro) scale [48], which is a reliable and extensively used tool to assess the methodological quality of randomized controlled trials. The total PEDro scores were assessed according to the satisfaction of the evaluation criteria for allocating and concealing participants, blinding participants and assessors, and providing sufficient statistical information. A total of 11 evaluation criteria were assessed; however, Criterion 1 was not included in the total PEDro score because it assesses external validity. Accordingly, a total score of 10 was obtained, which comprised the reporting of criterion 2–11.

## 3. Results

### 3.1. Study Selection and Characteristics

In the initial screening of the electronic databases, a total of 2837 studies (PubMed: 750, WoS: 428, Scopus: 1659) were identified, and an additional three papers were selected based on the reference list check. After removing duplicates, 2741 studies were retained. After the title and abstract examinations, 2658 articles were discarded (1965 were excluded after title examination and 693 were excluded after abstract examination). The full texts of the remaining 83 articles were assessed in more detail to determine eligibility. Each paper was read thoroughly and analyzed for study characteristics, participant information, description of the exercise intervention, and study outcomes. Of these articles, 72 did not meet the inclusion criteria; 11 articles met the inclusion criteria and were included in the systematic review. The process was summarized and presented in the PRISMA flow diagram (Figure 1).

Eleven studies from five different countries [Taiwan (n = 5), USA (n = 3), China (n = 1), Germany (1) and Japan (n = 1)] met the eligibility criteria. All included studies were randomized and controlled trials. Of the studies, 72.73% were within-subject in which repeated measures were administered, and 27.27% of the studies were between-subject design.

The control conditions of the included studies were reading [7,49,50,51,52], listening to an audio book [53], resting [54,55], sitting [56], given a task [57], and watching a video [58]. To isolate the acute effects of exercise, control conditions were chosen to allow comparison with activities of low cognitive and physical demand. Such controls allow for a clearer assessment of cognitive changes after exercising. The total number of participants varied from 16 [54] to 144 [57]; a total of 491 participants (220M; 271F) were included. Of the 11 studies, 9 of them included both males and females, whereas 2 of them included only males [49,58]. In two studies [49,51], based on baseline cardiorespiratory fitness (CRF) assessment, participants were divided into higher and lower fitness groups. Also, in two studies, various age groups were compared [57,58]. The exercise conditions included cycling [49,50,51,52,53,54,55,57] and walking on the treadmill [7,56,58]. The total duration of the exercise interventions varied from 10 min [54] to 45 min [50].

### 3.2. Quality and Completeness of Reporting

The detailed evaluation results obtained using the PEDro scale are presented in Table 1. The quality scores of the included 11 studies on the PEDro scale ranged from 3 to 6 (with a mean of 5.5 ± 1.18), with a maximum score of 10. All studies identified the eligibility criteria and provided statistical comparisons and valid measurements for at least one key outcome measure. However, 3 of the 11 studies reported baseline difference measures [51,53,54], and none reported the blinding of assessors. In addition, a key outcome measure for more than 85% of the subjects was reported in 8 of the 11 studies, and receiving exercise or the control condition as allocated for all subjects was reported in only 3 studies.

### 3.3. Executive Functions Outcome Variables

The primary outcomes were inhibition (n = 8), working memory (n = 2), and cognitive flexibility (n = 1). To assess inhibition, the Stroop [7,49,51,54,56,58], Flanker Test [55], and Saccadic Paradigm [52] were the most used tests. The N-back Task [53,57] was used for working memory; for cognitive flexibility, the task switching task [50] was used. Because of their controlled group post-test design, pre-intervention assessments were not reported in five studies [49,50,51,55,58].

#### 3.3.1. Inhibition (Interference)

The original version of the Stroop test was administered in one study [56] in which participants responded by saying the words; however, in the remaining studies, a keyboard or response pad was used.

Reaction Time (RT) in the congruent or the neutral (color) subtest, which refers to processing speed, was improved in four studies in MIAE condition, including 24 min at 60% HRR [56], 30 min at 50–60% HRR [49,52], 30 min at 60% HRR [51], and 30 min at 60–70 HRR [58]. Moreover, in two studies, participants were assigned into two (high and low fitness) groups based on ACSM guidelines [49,51]. There was a significant difference between the higher and lower fitness groups in that the higher fitness group had a quicker RT after the MIAE session in which participants cycled for 30 min at an intensity of 50–60% [49] and 60% HRR [51]. Furthermore, an improved accuracy rate in the congruent or the neutral (color) subtests was revealed in two studies after the MIAE condition, in which participants cycled for 30 min at 50–60% [51] and for 35 min at 15 RPE [55].

Quicker RT in the incongruent or interference subtest, which is related to EF and interference, was observed in five studies after MIAE, including 25 min at 65% of maximum heart rate (HRmax) [7], 30 min at 50–60% HRR [49], 30 min at 60% HRR [51], 10 min at ventilatory threshold (VT) [54], and 30 min at 60–70% HRR [58]. Additionally, the accuracy rate in the incongruent or the interference subtests was improved in two studies after the MIAE condition [51,55]. Additionally, an interference score (IS) (IS = incongruent RT or accuracy—congruent RT or accuracy) was calculated in three of the included studies, and a smaller IS was reported after the MIAE condition, including 30 min at 60% HRR [51], 10 min at VT [54], and 30 min at 60–70% HRR [58].

For the Saccadic Paradigm, there was a quicker RT in the antisaccade subtest, which is related to EF, and interference control was observed after the MIAE condition, including 30 min cycling at 50–55% HRR [52].

#### 3.3.2. Physiological Outcome Variables for Inhibition

The included studies simultaneously measured physiological variables during cognitive assessments using electroencephalogram (EEG) [49,58], near-infrared spectroscopy (NIRS) [7,54], and magnetic resonance imaging (MRI) [55] measurements. The event-related desynchronization (ERD) value in the prefrontal area comparison revealed that for lower alpha (8–10 Hz), a greater negative ERD value was produced in the MIAE condition than in the control condition (*p* < 0.001; 50.4%↓) [49]. Furthermore, when considering ERD values between 200 and 400 ms, there was greater negative ERD value production for upper alpha (11–13 Hz) in the exercise condition (*p* < 0.02; 50.6%↓). On the other hand, no significant interaction was observed between the condition and fitness levels for the ERD values [49].

Larger amplitudes were shown on the P3 component following the MIAE condition relative to the video-watching condition (*p* = 0.009; 9.4%↑); as well this, young participants had larger amplitudes than older adults (*p* = 0.011; 45.2%↑) [58]. Regarding the N450 component, larger amplitudes were observed following exercise (*p* = 0.005; 13.9%↑). In addition, during the congruent trials, similar amplitudes were observed between two groups, but not during the incongruent trials; young adults showed larger amplitudes relative to older adults (*p* = 0.044; 54.1%↑) [58].

Depending on fNIRS findings, a significantly greater oxygenated hemoglobin (Oxy-Hb) signal difference was observed in the MIAE condition than in the control condition in R-FPA (*p* < 0.01; 131%↑) [54]. In addition, there was a significant coincidence between RT and R-FPA activation (12/16 enhancement in RT with activation; *p* < 0.05). Similarly, greater oxygenation levels were revealed in the MIAE condition than in the control condition in L-DLPFC (*p* = 0.031; 400%↑) and R-DLPFC (*p* = 0.031; 207.7%↑) in the executive task [7].

Lastly, in one study [55], the interference score was calculated (incongruent—congruent trials) following the administration of the Flanker task during an fMRI scan. For the congruent trials and IS calculations, significantly greater activation was found in the left inferior frontal gyrus (*p* = 0.020; 120.0%↑) and two regions of the left inferior parietal lobule (*p* = 0.0007; 3400.0%↑; *p* = 0.0004; 1100.0%↑) in the MIAE condition than under the control condition.

#### 3.3.3. Cognitive Flexibility (Shifting)

Cognitive flexibility was assessed using a task-switching paradigm. For global switching in heterogeneous conditions, shorter RT was observed in the 20 min MIAE session compared with the control (*p* < 0.02; 6.4%↓) and shorter (10 min) MIAE session (*p* < 0.05; 6.2%↓) [50]. However, no significant differences in RT were observed in the homogeneous condition, neither was accuracy. Regarding RT for local switching, results were nearly the same as global switching in that RT after 20 min MIAE was quicker than the control (*p* < 0.03; 6.5%↓) and 10 min MIAE session (*p* < 0.03; 6.5%↓).

#### 3.3.4. Working Memory

Two studies [57] investigated the acute effects of aerobic exercise on working memory in older adults with a moderation in age following acute exercise on cognitive performance and affective experience. Regarding the accuracy of 2-back performance, Hogan et al. [57] suggested that after 15 min of moderate-intensity aerobic exercise, there was no significant effect of age, condition, or interaction between the two. However, 2-back performance after exercise was associated with a quicker RT compared with the control condition (*p* = 0.014; 8.5%↑). On the contrary, Stute, Hudl, Stojan, and Voelcker-Rehage [53] reported no significant change in RT between the exercise and control groups. For accuracy, no significant difference was observed between the conditions in the included studies [53,57]. These effects were not associated with age or gender; therefore, the effect of moderate exercise on working memory was relatively consistent across the sampled age range.

#### 3.3.5. Physiological Outcome Variables for Working Memory

Stute, Hudl, Stojan, and Voelcker-Rehage [53] simultaneously measured physiological variables during cognitive assessments using fNIRS. For the statistical analysis, the researchers calculated the hemoglobin difference between the oxygenated and deoxygenated concentration changes in the cortex. The findings of the fNIRS analysis indicated no significant difference in hemoglobin levels between the groups in either the hemisphere or the frontal and parietal regions (Table 2).

## 4. Discussion

The purpose of this systematic review was to examine the acute effects of moderate-intensity exercise on core EF (inhibition, cognitive flexibility, and working memory) in healthy older adults. Overall, in 10 of the 11 included studies, significant improvements in at least one variable (RT or accuracy of congruent, incongruent, neutral or the interference score for inhibition, global or local switch cost for flexibility, and RT or accuracy for working memory task) were reported. The included studies were evaluated as medium quality based on the PEDro scale. The highest magnitude of changes was observed (d = 0.47; 7.4%↓) on inhibition where participants exercised for 30 min between 50% and 60% HRR [49], and the shortest exercise intervention with a positive cognitive outcome was 10 min (3 min warm-up) at VT, and the cognitive test was administered after 15 min of exercise cessation in both studies. In contrast, minor changes were detected in working memory (d = 0.02; 0.5%↑) on a 23 min MIAE intervention (including 5 min warm-up and 3 min cool-down) at 50% HRR [57], and no changes were reported for working memory in 15 min MIAE intervention.

### 4.1. Acute Effects of MIAE on Inhibition

The improved processing speed after MIAE [56] is consistent with the literature [28,31,59] to some degree. A shorter RT after 20 min of MIAE was observed in late middle-aged adults [31], not only for the neutral trials but also for the incongruent trials [28,31,59]. Exercise duration, intensity, and testing time are among the main moderators of acute exercise-induced effects on cognitive function [42]. These parameters are nearly the same as those reported for enhanced processing speed and inhibition [7,31,49,51,52,58,59]. Therefore, exercise duration, intensity, and testing time cannot explain the differences observed in the rest of the literature. Because the time course of MIAE on inhibition was studied by Barella et al. [56], cognitive tests were administered several times in succession. There is evidence that multiple repetitions of performing an action or even a simple test may cause both mental and physical fatigue that impairs performance [60]. Moreover, repeated tests may result in changes in motivation, mood, or arousal [61]. Therefore, additional testing of these moderators may improve the understanding of the effects of acute MIAE in older adults.

Acute MIAE enhances processing speed and inhibition in older adults [49,51,52,58] which is parallel to studies conducted in adolescents [29] and young adults [32,36]. In addition, depending on the CRF of the participants, the higher fitness groups benefit more from MIAE than the lower fitness groups in older adults [49,51] which is not compatible with studies on young adults [35,62]. Compared with participants with low CRF, older adults with higher CRF levels may maintain healthier brain structures or better density (e.g., white and gray matter). Therefore, it can be said that older adults with higher fitness levels benefit more from acute MIAE.

Studies in which the interference score was calculated [7,54,58] are also in line with studies conducted on young adults [30,34,37,39]. Acute MIAE has the potential to enhance inhibition, but not for basic processing speed, in older adults [7,54], which is supported by studies that suggested that the effects of MIAE are likely to be more sensitive for demanding tasks than automatic, effortless tasks [63,64]. In addition, it is suggested that 7 min of exercise at VT (app. 50% HRR) has beneficial effects on inhibition, but not on processing speed, in older adults [54]. This result is consistent with a study conducted on young adults who performed low-intensity exercise for 10 min [8] but inconsistent with the suggestion of no enhancement after 10 min of MIAE in late middle-aged adults [31]. The VT and anaerobic threshold are correlated [65], and exercises at the VT level cause anaerobic threshold raises. Therefore, this may have positively affected cognitive functions more than the calculated HRR exercises. On the other hand, it is difficult to determine VT because a spirometer is required, which limits its practical application. However, when the sweet spot for positive cognitive outcomes is considered, VT exercises must be examined in future studies. In addition, while comparing these studies for control conditions, control participants only rested in the study by Byun et al. [8] and Hyodo et al. [54]. However, in the study conducted by Chen, Yan, Chen, Kuan, Wei, Hung and Chang [31], control participants were allowed to read a book that may enhance arousal or motivation more than resting, which may affect post-measurements. Therefore, efforts to control motivation, feelings, affect, or arousal can provide more controlled control conditions. Measuring these variables in both the control and intervention participants may also enhance the quality of the research. Only one study performed this measurement using a self-assessment manikin [66] to assess subjective effects among the studies included in this review [55]. However, such measurements are based on self-reporting, and people may not report their real situation to the tests.

Regarding accuracy, participants in the MIAE condition have been reported to perform more accurately in congruent and incongruent trials [51,55], which is inconsistent with other studies [7,30,54,58] that reported no significant differences between the MIAE and control conditions. In one study [51], the familiarization of the test procedure ended when participants achieved 85% accuracy, which may have resulted in more learning effects. Moreover, previous studies have selected different lengths for the response window for the Stroop test: 1000 ms [49,51], 1500 ms [58], 2000 ms [7,54], and 3000 ms [56]. Therefore, the familiarization procedure and the response window may moderate accuracy. In particular, the adaptation of a longer response window may conceal positive effects on accuracy due to a ceiling effect (e.g., too high accuracy). Also, the effects of exercise may be task and time dependent. Although Chang et al. [49], Chu et al. [51], and Won et al. [55] used nearly the same intensity and duration of exercise, the difference may occur because of the administration time of the different cognitive tests. It can be speculated that the positive effects of following MIAE on RT and accuracy occur immediately, and only the effects on RT are maintained for longer in older adults. Therefore, more dose–response and time-course studies with different inhibition tasks are needed to clarify the effects of MIAE on RT and accuracy in older adults.

#### Physiological Outcome Variables for Inhibition

It has been suggested that alpha ERD is positively associated with attentional allocation for anticipation [67] and reflects a top-down process [68]. Therefore, larger alpha ERD values after MIAE, in alignment with Chaire et al. [38], suggested that exercise may provide neural resources for top-down processes and attentional allocation to enhance cognition in young adults [49]. In addition, increased higher alpha ERD between 200 and 400 ms revealed that MIAE may affect the task specifically. The tasks required to allocate attention that is crucial between 200 and 400 ms may be vulnerable to exercise. Therefore, future studies investigating the task-specific effects of MIAE in older adults should be encouraged. On the other hand, the higher and lower CRF groups showed similar ERD values in the cognitive test. This result contradicts studies that suggested that participants with higher CRF levels showed “less” cortical activation (weaker ERD) in task-relevant brain areas than lower CRF level counterparts in young adults [69,70], which supports the neural efficiency theory [71,72].

Based on ERP studies, the P3 amplitude reflects the amount of attention allocated to the processing of target stimuli; on the other hand, P3 latency reflects processing speed [37,73]. The observation of larger P3 amplitudes in the MIAE condition, regardless of task demands [58], in agreement with Kao et al. [39], reported increased P3 amplitude after moderate exercise in young adults.

The N450 component is a conflict marker that larger N450 amplitudes reflect monitoring processes involved in conflict detection [74]. The findings of Hsieh et al. [58] were supported by Chang et al. [37] that significantly smaller N450 amplitudes following MIAE were observed in young adults. In addition, young adults exhibited larger N450 amplitudes in the incongruent trials, and larger P3 amplitudes also supported the findings that young adults had faster RTs than older adults. Therefore, enhanced inhibition following acute MIAE, at least in part, is a result of the efficient allocation of attention and conflict monitoring signified by P3 and N450 components, respectively [37].

Several functional neuroimaging studies have suggested that the DLPFC [6], VLPFC [7], FPA [8], IFG [9,10], and IPL [12] are involved in complex cognitive processes related to brain region activation. After the MIAE session, a Stroop interference-based allocation of cortical activation increment in the R-FPA was observed [54]. Moreover, this increased cortical activation significantly coincided with the RT, which indicates that the R-FPA can be one of the neural bases of improved cognitive performance following exercise in older adults. On the other hand, it was revealed that the oxy-Hb level bilaterally increased in DLPFC and VLPFC following MIAE [7]. These results are supported in part by studies conducted on young adults [6,8]. Greater oxy-Hb levels after mild exercise in the L-FPA and L-DLPC [8] was in alignment with improved L-DLPC activation after MIAE [6]. Moreover, the increased activations coincided with RT in both studies [6,8]. Therefore, exercise-induced activation of the cortex may depend on participant characteristics (e.g., age, fitness level) and exercise intervention (e.g., duration, type, intensity). With age, reduced lateralization was reported, whereas older participants showed bilateral activation during tasks, whereas younger participants showed that two activated lateral areas have separate roles [75]. In contrast, other studies have suggested a negative relationship between PFC activation and cognitive performance [76] and no relationship between CBF oxy-Hb levels and cognitive performance [77]. Taken together, these findings suggest that there is no consensus on exercise-induced activation in the PFC, and more studies are needed to understand which task-related area of the cortex is activated and involved in inhibition in older adults.

Neuroimaging studies have also focused on the relationship between IFG and inhibitory control. It has been suggested that the collectivity of the left IFG is vital for the successful execution of inhibitory control upon motor responses [9,10], and left IPL is important for recruiting the allocation of more attentional resources [12]. In light of these findings, greater activation of the left IFG as well as in left IPL during the Flanker task following MIAE was reported [55]. As a result, greater recruitment of both the IFG and IPL following MIAE may be a neural network to resolve interference and enhance attentional resources to focus on the target cues.

### 4.2. The Acute Effects of MIAE on Cognitive Flexibility

Longer durations of at least 20 min of MIAE enhance task switching; durations of 45 min were also suggested [50]. This result was in line with previous findings on young adults [30]. In addition, the reviews suggested that exercise for at least 20 min [78] or between 20 and 60 min [59] could enhance cognitive function, which is supported by the finding that a 20 min MIAE session improved heterogeneous condition performance of global switching and non-switch and switch trials of local switching [50]. Considering that heterogeneous conditions are the most cognitively demanding subtest of the task, exercise may be more useful for demanding tasks than effortless tasks [63,64]. In addition, the examination of the physically active older adults with high CRF levels [50] may affect the study results [79]. The study conducted on young adults suggested that after acute MIAE, RTs in the switching task increased; however, only participants with high CRF benefited from MIAE because of lower switching costs [40]. As mentioned earlier, participants with higher CRF levels cognitively benefit more from acute exercise than those with lower CRF levels in older adults [49,51]. Because CRF levels are likely to become more important for cognitive outcomes of exercise with age [80], more studies with various CRF levels are needed to understand MIAE effects with the moderation of CRF on cognition in various age groups.

Lastly, it is suggested that 10 min of moderate exercise is too short to affect cognitive flexibility in physically active older adults [50]. Although most of the conducted studies prescribed 20 min MIAE for cognitive benefit and ACSM guidelines for general benefits [81], the dose–response relationship for the duration of MIAE and cognitive outcome is not clearly understood in healthy older adults. Therefore, more studies investigating the dose–response relationship among older adults are needed.

### 4.3. The Acute Effects of MIAE on Working Memory

It has been suggested that a single bout of MIAE for 15 min is beneficial for working memory, independent of age [57], which is compatible with the study of Tsujii et al. [82] in which 10 min of light exercise resulted in improved working memory in older adults. In fact, several studies [83,84,85] have suggested that physical exercise has positive effects on cognition enhancement and protection of cognition against age-related decline. However, there is no consensus on the effects of aerobic exercise on working memory, and some of the conducted studies on younger age groups reported no changes in working memory after aerobic exercise on adolescents [86], young adults [87,88,89], and older adults [53]. On the contrary, some studies have observed improved working memory after exercise in preadolescent children [90,91], young adults [92,93], and older adults [57]. Because WM is not stable [94], the acute factors, such as emotional state, malleability (e.g., stress, sleep), and chronic factors like intelligence, age, and personality, can affect performance. Therefore, researchers should consider the administration of additional measures to control these factors. In addition, more studies are needed to understand the mechanisms and potential effects of the acute MIAE on working memory.

#### Physiological Outcome Variables for Working Memory

Stute, Hudl, Stojan, and Voelcker-Rehage [53] reported no difference in the calculated hemoglobin difference between the exercise and control groups. They also reported higher hemoglobin difference levels in parietal regions than frontal regions. These findings contradict those of the fNIRS studies on inhibition mentioned above, and further studies are needed to elucidate this issue in the elderly population, as this is the only study in the literature that has been studied acutely.

### 4.4. Study Limitations and Future Directions

First, the participants of the included studies were healthy older adults. In fact, some of the studies included not only healthy but also physically active older adults. As seen in this review, CRF can be a moderator of the outcome of the exercise condition. It has been reported that the effects of acute MIAE on cognitive function may differ between age groups. For example, rapid gains in reaction time and inhibition have been observed in young adults after a short period of MIAE [42]. However, it has been suggested that the duration or intensity of exercise may need to exceed a certain threshold for cognitive gains to occur in middle-aged and older age groups [50,54]. These differences may be explained by age-related structural and functional changes in the brain, as well as variations in CRF levels and the possible presence of chronic diseases. Therefore, future research should investigate the effect of exercise on cognitive functions more precisely by developing protocols specific to different age groups and interpreting the results obtained in the light of age-related neurophysiological changes. In fact, participants with cognitive impairment may have functional dissimilarities or distinguished neuropathology compared with healthy older adults. Therefore, the results of the included studies and this review cannot be directly generalized to older adults. Moreover, the literature suggests that CRF levels may play a critical role in the cognitive response to the MIAE [49,51]. In particular, older adults with higher CRF levels have been reported to show greater improvements in executive function components such as inhibition and cognitive flexibility following MIAE [49,51]. In contrast, the benefit was more limited in groups with lower CRF levels [35,62], suggesting that exercise-induced cognitive gains in older adults may differ according to personal fitness levels. This may be explained by the fact that individuals with higher CRF levels have greater cerebrovascular reserve or brain plasticity [79,80]. Therefore, it is recommended that further research should use larger samples and stratified designs, taking into account the different CRF levels of older groups.

Second, ACSM recommends not only aerobic training but also strength training for older adults. However, in this review, only the effects of MIAE on EF were examined. Therefore, more studies are needed to determine how cognitive outcomes are affected by interacting with each other, mainly the intensity (low-medium-high), duration (short-long), type (endurance, strength, balance, and flexibility), and mode (closed-open skill) of the training, and the time of administration of cognitive tests.

Third, although this study included randomized and controlled trials, the mean score of the studies based on the PEDro scale was 5.5, which can be evaluated as medium quality. Therefore, it is clear that more methodologically robust studies are needed. In this manner, it is nearly impossible for participants to be blinded to exercise or control conditions, but assessors of cognitive tests can be blinded. Therefore, researchers should consider conducting at least single-blinded studies, which will increase the methodological quality. The absence of a double-blind application and the expectation that the placebo effect cannot be eliminated may cause bias; therefore, this is another issue to be considered when generalizing conclusions from such studies. Also, it is recommended to design experiments with standardized measurements (e.g., a standardized battery of cognitive tests) and control conditions to control factors that can affect performance, such as motivation and arousal. In addition, most of the studies in the review used common control conditions with light physical and cognitive load, such as reading (five studies) and resting (two studies), while the remaining studies used different resting conditions (e.g., sitting quietly, listening to an audiobook). This variability in control conditions limits the generalizability of the findings, but it should be noted that this methodological diversity also provides an important data contribution.

Fourth, the within-subject design, preferred by the majority of studies included in the systematic review, has the advantage of providing greater statistical power by minimizing between-subject variability. It is important to note that although this method minimizes bias that may arise from between-group differences, it is a design that is more sensitive to transient effects such as learning, fatigue, or sequence effects.

Fifth, the Stroop test and Flanker task reflect the only interference aspect of inhibition. Therefore, one must be aware of the generalization of the results. In addition, it can be said that set-shifting and task switching are different subdomains of cognitive flexibility in the type of conflict. Set shifting refers to shifting attention between different features of the same stimulus to follow given instructions. On the other hand, task switching involves switching between different tasks according to given instructions that involve different stimuli [95]. Therefore, studies that assess inhibition related to motor suppression and cognitive flexibility with set shifting are needed to broaden the knowledge of these EF domains. Moreover, although there is a tendency in the literature to examine different cognitive domains, it is recommended that future studies should use similar test batteries with similar control conditions to increase comparability.

Sixth, the reported differences in neuroimaging findings may be primarily due to demographic characteristics of the participants, such as age group, CRF level and exercise history. The intensity, duration, and type of exercise may modulate brain activity in different ways, leading to inconsistent results. Also, the different spatial and temporal resolution characteristics of different neuroimaging techniques, such as fNIRS, EEG, and fMRI, change the way brain activity is captured and interpreted. Therefore, this methodological and sampling level diversity makes it difficult to directly compare neuroimaging results, leading to discrepancies between findings.

Finally, this review focuses on only the core aspects of EF, and the findings may not be generalized to other aspects of cognition. When considering the publishing years of the studies included in this review, it can be concluded that the interest in this field is increasing continuously. Therefore, it is clear that more studies are needed to clarify the neural mechanisms underlying acute MIAE in healthy adults.

## 5. Conclusions

The present systematic review demonstrated that acute MIAE had positive effects on core EF in healthy older adults. Overall, 10 of the 11 included studies reported an enhancement of at least one of the core EF variables following the MIAE session. In particular, the duration, intensity, and type of exercise protocol are important variables in cognitive performance outcomes. For example, 30 min of moderate-intensity exercise (50–60% HRR) has been reported to produce the greatest change in inhibition performance [49]. On the other hand, exercise of up to 10 min duration, but performed at the VT, has been reported to have beneficial effects, particularly on reaction time [54]. Although it has been suggested that cognitive benefits increase with increasing exercise intensity, some studies with very short protocols (≤10 min) failed to show significant results [30], while others showed only partial improvements in certain domains (e.g., processing speed) [8]. In addition, the fact that different exercise modes, such as bicycle ergometer or treadmill, affect the cardiorespiratory response and the neurological arousal level differently may lead to different results on cognitive performance when exercises are performed for the same duration. Therefore, the duration, intensity and mode of exercise should be evaluated as a whole, and more research is needed to determine the optimal levels of these parameters, especially in older adults. In addition, participants with higher CRF levels benefit more from acute MIAE. Therefore, it is crucial for older adults to maintain CRF at higher levels to sustain cognitively healthy functioning. The improved inhibition following MIAE was associated with increased attention allocation and enhanced conflict monitoring and detection. Moreover, greater R-FPA in the left IFG and left IPL activation is associated with improved inhibition performance following MIAE. However, there are contradictory findings on exercise-induced activated areas of the PFC in older adults. Moreover, it is recommended that assessor blinding, standardization of cognitive testing protocols and control conditions, and measurement and reporting of potential confounding variables such as motivation and mood be ensured to improve the methodological quality of future studies. In addition, the use of neuroimaging and biomarkers may provide more comprehensive data to help explain the potential benefits of MIAE on cognitive function. In this way, both the exercise protocol and participant characteristics (e.g., CRF level, age) will be controlled for in detail, allowing for clearer and more generalizable results in future studies. Lastly, although the increasing number of studies investigating the effects of MIAE on EF in older adults has been growing over the last decade, there are still limited studies especially examining the effects of MIAE on working memory and cognitive flexibility in older adults.

## Figures and Tables

**Table 1:** Quality assessment of the included studies.

Study	C 1	C 2	C 3	C 4	C 5	C 6	C 7	C 8	C 9	C 10	C 11	Total
Barella et al. [56]	Y	Y	N	Y	N	N	N	Y	Y	Y	Y	6
Chang et al. [49]	Y	Y	N	Y	N	N	N	Y	N	Y	Y	5
Chen et al. [50]	N	Y	N	Y	N	N	N	Y	Y	Y	Y	6
Chu et al. [51]	N	Y	N	N	N	N	N	N	N	Y	Y	3
Hogan et al. [57]	N	Y	N	Y	N	N	N	Y	Y	Y	Y	6
Hsieh et al. [58]	Y	Y	N	Y	N	N	N	N	N	Y	Y	4
Hyodo et al. [54]	Y	Y	N	N	N	N	N	N	N	Y	Y	3
Ji et al. [7]	Y	Y	N	Y	N	N	N	Y	N	Y	Y	5
Stute et al. [53]	Y	Y	N	Y	N	N	N	Y	Y	Y	Y	6
Tsai et al. [52]	Y	Y	N	Y	N	N	N	Y	Y	Y	Y	6
Won et al. [55]	Y	Y	N	Y	N	N	N	Y	N	Y	Y	5

NOTE. C 1: Eligibility criteria were specified (not included in the total score). C 2: Subjects were randomly allocated to groups. C 3: Allocation was concealed. C 4: The groups were similar at baseline regarding the most important prognostic indicators. C 5: There was blinding of all subjects. C 6: There was blinding of all therapists who administered the therapy. C 7: There was blinding of all assessors who measured at least one key outcome. C 8: Measures of at least one key outcome were obtained from more than 85% of the subjects initially allocated to groups. C 9: All subjects for whom outcome measures were available received the treatment or control condition as allocated. C 10: The results of between-group statistical comparisons are reported for at least one key outcome. C 11: The study provides both point measures and variability measures for at least one key outcome. Abbreviations: C = criterion; N = no; Y = yes.

**Table 2:** Summary of the included studies.

Author(s)	Study Design	Participants		Exercise Intervention			Cognitive Outcomes		
		Age, Years (SD)	n	Type	Duration	Intensity	Task	Domain	Timing of Test
Barella et al. [56] (2010)	Randomized Controlled Trial	69.5 ± 8.3	CG: 20 EG: 20 (32F, 8M)	CG: Sitting at the end of the treadmill EG: Treadmill walking	CG: 25 min. EG: 25 Min (including 5 min WU)	CG: NA EG: 60% HRR	The Stroop test	Inhibition	Immediately after 5, 10, 15, 20, 30, 45, 60, 75, 90, 105 and 120 min after
Chang et al. [49] (2015)	Counter-balanced randomized and controlled group post-test	63.10 ± 2.89	LFG: 21 HFG: 21 (42M)	CG: Reading a book EG: Cycling	CG: 30 min EG: 30 Min (including 5 min WU and 5 min CD)	CG: NA EG: 50% to 60% HRR	Computerized version of the Stroop test	Inhibition	15 Min after
Chen et al. [50] (2018)	Counter-balanced randomized and controlled group post-test	57.67 ± 5.06	Total: 45 (26F, 19M)	CG: Reading a book EG: Cycling	CG: 30 Min. EG1: 20 Min; EG2: 30 Min; EG3: 55 min. (All EC including 5 min WU and 5 min CD)	CG: NA EG: 65% to 70% HRR	The task switching task	Cognitive Flexibility	Immediately after
Chu et al. [51] (2015)	Counter-balanced randomized and controlled group post-test	HFG: 63.8 ± 2.3 LFG: 64.9 ± 4.0	HFG: 22 LFG: 24 (22F, 24M)	CG: Reading a book EG: Cycling	CG: 30 Min EG: 30 Min (including 5 min WU and 5 min CD)	CG: NA EG: 60% HRR	Computerized version of the Stroop test	Inhibition	<5 min after
Hogan et al. [57] (2013)	Stratified randomized and controlled	YG: 19–39 years MAG: 40–64 years OG: 65+	Total: 144 (73FM, 71M)	CG: Subjective picture quality rating EG: Cycling	CG: 15 to 25 min EG: 23 Min (including 5 min WU and 3 min CD)	CG: NA EG: 50% HRR	N-back task	Working Memory	Immediately after
Hsieh et al. [58] (2018)	Counter-balanced randomized and controlled group post-test	YG: 24.0 ± 3.1 OG: 70.0 ± 3.3	YG: 24 (24M) OG: 20 (20M)	CG: Watching videos EG: Treadmill walking	CG: 30 Min EG: 30 Min (including 5 min WU and 5 min CD)	CG: NA EG: 60–70% HRR	Modified Stroop Color-Word Test	Inhibition	15 Min after
Hyodo et al. [54] (2012)	Counterbalanced, randomized, and controlled	69.3 ± 3.5	Total: 16 (3F, 13M)	CG: Resting EG: Cycling	CG: 10 Min EG: 10 Min (including 3 min WU)	CG: NA EG: at VT (app. 50% VO2max)	The color-word matching Stroop task	Inhibition	15 Min. after
Stute et. al. [53] (2020)	Counterbalanced, randomized, and controlled	69.18 ± 3.92	Total: 42 (21F, 21M)	CG: Listening to an audio book EG: Cycling	CG: 15 min EG: 10 min cycling	CG: NA EG: 50% VO2max	N-back task	Working Memory	15, 30, and 45 min after
Tsai et. al. [52] (2021)	Counterbalanced, randomized, and controlled	61.15 ± 4.43	Total: 20 (10F, 10M)	CG: Reading a book EG: Cycling	CG: 30 Min EG: 30 Min (including 4 min WU and 2 min CD)	CG: NA EG: 50% to 55% HRR	Saccadic Paradigm	Inhibition	3–5 min after
Ji et al. [7] (2019)	Counterbalanced, randomized, and controlled	65.60 ± 1.32	Total: 20 (9F, 11M)	CG: Reading a book EG: Treadmill walking; cognitive exercise; cognitive + treadmill walking	CG: 25 min. EG: 25 Min (including 5 min WU and 5 min CD)	CG: NA EG: 65% HRmax	The Modified Stroop test	Inhibition	Immediately after
Won et al. [55] (2019)	Counter-balanced randomized controlled group post-test	66.2 ± 7.3	Total: 32 (24F, 8M)	CG: Resting EG: Cycling	CG: 35 Min EG: 35 Min (including 5 min WU; 5 min CD and 5 min recovery)	CG: NA EG: RPE of 15	Flanker Task	Inhibition	30–40 Min after

Abbreviations: CD = cool down; CG = control group; EG = exercise group; HFG = high-fitness group; LFG = low-fitness group; MAG = middle-aged group; NA = not available; OG = old group; WU = warm-up group; YG = young group.

## Supplementary Materials

The following supporting information can be downloaded at: https://www.mdpi.com/article/10.3390/life15020230/s1, File S1. PRISMA Checklist.
